# Providers’ perceptions of communication and women’s autonomy during childbirth: a mixed methods study in Kenya

**DOI:** 10.1186/s12978-020-0909-0

**Published:** 2020-06-03

**Authors:** Patience A. Afulani, Laura Buback, Ann Marie Kelly, Leah Kirumbi, Craig R. Cohen, Audrey Lyndon

**Affiliations:** 1 School of Medicine, University of California, San Francisco (UCSF), 550 16th St, 3rd Floor, San Francisco, CA 94158 USA; 2 UCSF Institute for Global Health Sciences, San Francisco, USA; 3 Sidney Kimmel Medical College, Thomas Jefferson University, Philadelphia, USA; 4 Kenya Medical Research Institute, Nairobi, Kenya; 5 Rory Meyers College of Nursing, New York University, New York, USA

**Keywords:** Communication, Autonomy, Person-centered maternity care, Respectful maternity, Quality of care, Person-centered care, Patient-provider interactions

## Abstract

**Background:**

Effective communication and respect for women’s autonomy are critical components of person-centered care. Yet, there is limited evidence in low-resource settings on providers’ perceptions of the importance and extent of communication and women’s autonomy during childbirth. Similarly, few studies have assessed the potential barriers to effective communication and maintenance of women’s autonomy during childbirth. We sought to bridge these gaps.

**Methods:**

Data are from a mixed-methods study in Migori County in Western Kenya with 49 maternity providers (32 clinical and 17 non-clinical). Providers were asked structured questions on various aspects of communication and autonomy followed by open ended questions on why certain practices were performed or not. We conducted descriptive analysis of the quantitative data and thematic analysis of the qualitative data.

**Results:**

Despite acknowledging the importance of various aspects of communication and women’s autonomy, providers reported incidences of poor communication and lack of respect for women’s autonomy: 57% of respondents reported that providers never introduce themselves to women and 38% reported that women are never able to be in the birthing position of their choice. Also, 33% of providers reported that they did not always explain why they are doing exams or procedures and 73% reported that women were not always asked for permission before exams or procedures. The reasons for lack of communication and autonomy fall under three themes with several sub-themes: (1) work environment—perceived lack of time, language barriers, stress and burnout, and facility culture; (2) provider knowledge, intentions, and assumptions—inadequate provider knowledge and skill, forgetfulness and unconscious behaviors, self-protection and comfort, and assumptions about women’s knowledge and expectations; and (3) women’s ability to demand or command effective communication and respect for their autonomy—women’s lack of participation, women’s empowerment and provider bias.

**Conclusions:**

Most providers recognize the importance of various aspects of communication and women’s autonomy, but they fail to provide it for various reasons. To improve communication and autonomy, we need to address the different factors that negatively affect providers’ interactions with women.

## Plain English summary

Women value effective communication and respect for their autonomy during childbirth. The World Health Organization has recommended certain practices as minimum standards for effective communication and respect for women’s autonomy during childbirth. Yet, little is known about providers perceptions of the importance of these practices, the extent to which providers engage in these practices, and the barriers to effective communication and maintenance of women’s autonomy during childbirth. We used data from interviews with 49 maternity providers in Migori County in Western Kenya to understand these issues. We found that despite awareness of the importance of various practices related to communication and women’s autonomy, communication was sometimes poor, with a lack of respect for women’s autonomy. For example, 57% of respondents reported that providers never introduce themselves to women and 38% reported that women are never able to be in the birthing position of their choice. Also, 33% of providers reported that they did not always explain why they are doing exams or procedures and 73% reported that women were not always asked for permission before exams or procedures. The reasons providers gave for not engaging in these practices included perceived lack of time, language barriers, stress and burnout, facility culture, inadequate provider knowledge and skill, forgetfulness and unconscious behaviors, self-protection and comfort, assumptions about women’s knowledge and expectations***,*** women’s lack of participation, and provider bias. To improve communication and autonomy, we need to implement interventions to address the different factors that negatively affect providers’ interactions with women.

## Background

High quality care is critical to sustain global efforts to improve maternal and neonatal outcomes [1–3]. The World Health Organization (WHO) Framework for Maternal and Newborn Health highlights two aspects of quality of care: “Provision of Care” and “Experience of Care” [4, 5]. “Provision of Care” captures the technical elements of care, while “Experience of Care” captures the interpersonal elements of care [6]. “Experience of Care” also emphasizes person-centered care—care that is respectful and responsive to user preferences, values, and needs [7, 8].. Communication is a key element of experience of care and a critical component of person-centered care and health system responsiveness [7, 9, 10].

Effective communication requires that providers clearly explain to patients and family the nature of their condition, details of treatment, and available treatment options [9]. It also requires that providers establish rapport, communicate respect, consider the patient’s perspective, and demonstrate empathy [11]. Patient-centered communication enables providers to be more engaged with patients by listening to what they have to say, asking questions, and being sensitive to their concerns [12]. Closely linked to communication is *autonomy,* which is respect for patients’ views of what is appropriate and allowing them to make informed choices [9]. Effective communication facilitates patient understanding and involvement in decision-making. It is therefore a crucial component of patient autonomy.

Effective provider-patient communication is associated with higher patient satisfaction with care and safety [13, 14]. Patient-centered communication is associated with improvements in medication adherence, emotional health, physiologic measures (i.e., blood pressure and blood sugar levels), pain control, fewer diagnostic tests and referrals, symptom resolution, functional status, and lower mortality [11, 13, 15]. While there is little evidence on how communication and autonomy affect maternal and neonatal outcomes, there *is* considerable evidence that women value effective communication and respect for their autonomy [16–19].

The *WHO recommendations for Intrapartum care for a positive childbirth experience* include minimum standards of effective communication and autonomy. These include: providers introducing themselves and addressing women by name; offering clear and concise medical information; responding to women’s needs, preferences, and questions; supporting women to understand they have choices; ensuring explanation of procedures and consent; ensuring women are aware of mechanisms for addressing complaints, and positively interacting with women’s companions [19]. Yet, several studies on women’s experiences highlight poor communication and lack of respect for their autonomy during childbirth [16, 17, 20–25].. A recent study in four settings across Kenya, Ghana, and India found that “communication and autonomy” was the domain of person-centered maternity care (PCMC) that was consistently poor across the settings [26].

Little is known about providers’ perceptions of the WHO recommendations on communication and autonomy during childbirth in low-resource settings [27]. In addition, prior studies have shown that low quality of care can occur even when health care practitioners have appropriate knowledge. This leads to a “know-do” gap where providers’ actions diverge from what they know they should do [28, 29]. Thus, it is important to understand the factors that might underlie the “know-do” gaps in communication and autonomy in order to develop effective and practical strategies for improvement.

This study seeks to extend the literature on communication and women’s autonomy during childbirth using data from clinical and non-clinical (support staff) maternity providers. Prior studies in the setting have shown that both types of providers play a key role in women’s birth experiences [16, 30]. Our research questions are: (1) what are providers’ perceptions of the importance of communication and women’s autonomy during childbirth; (2) what are providers’ perceptions of the extent of communication and women’s autonomy during childbirth provided by themselves and other providers in their facilities; and (3) what are the barriers to effective communication and maintenance of women’s autonomy during childbirth?

## Methods

We used a convergent mixed-methods design to address our research questions. Data for this analysis are from a larger mixed methods study on community perceptions of quality of maternity care in Migori county, Kenya, which is described in detail elsewhere [31, 32]. Migori county is located in western Kenya and has eight sub-counties, each of which has a sub-county hospital in addition to several health centers. It has a population of about one million and an estimated 40,000 births annually [33]. It is served by 32 nurses, 19 clinical officers, and four doctors, per 100,000 people [34]. Maternal and child health indicators are generally lower in this county than in other counties in Kenya: it has an estimated maternal mortality ratio of 673 deaths per 100,000 live births compared to the national average of 495; and about 53% of births in the County occur in health facilities, compared to the national average of 61% [35].

In this paper, we use data from 49 clinical and non-clinical providers working in maternity units across all sub-counties of Migori. Interviews occurred in October and November 2016. Two Kenyan female research assistants with college degrees were trained by the first author to conduct the interviews.

Providers were purposefully sampled from 18 facilities selected for an intrapartum quality improvement project based on their relatively higher volume of births. With permission from the county and facility leaders, the research assistants approached individual providers in maternity units, informed them about the study and invited them to participate. All agreed to participate and interviews occurred at only one time point. The research assistants conducted the interviews in private using a questionnaire with both closed and open-ended questions. The research team comprised a physician with a PhD in public Health and mixed-methods training (PA), two master’s level prepared researchers (LB and AK), two obstetrician gynecologists (LK and CC), and a PhD-prepared nurse scientist with maternity care and qualitative methods expertise (AL). Five team members were female (PA, LB, AK, LK, and AL), and one was male (CC) (COREQ checklist in Additional file 1).

Communication and autonomy were operationalized by items in the communication and autonomy sub-scale of the PCMC scale, which captures the WHO minimum standards [36]. Providers were asked to assess the relative frequency at which certain practices related to PCMC occurred using closed questions with structured responses. For selected questions, providers were asked to make this assessment based on what practices they believe generally occurred in their facilities, as well as what practices they engaged in themselves. They were also asked to assess the importance of certain practices by stating if it was *not important*, *somewhat important*, or *very important*. Each closed-ended question was followed by open-ended questions regarding their responses to assess why certain practices were done or not done and why participants thought certain aspects of care were important or not. The interviews were conducted in English, Swahili or Luo in private spaces in each health facility. Each interview lasted about an hour. The structured responses were directly entered into the REDCap application on a tablet [37]. Interviews were audio-recorded and transcribed (with simultaneous translation where necessary). All participants provided written informed consent. The study was approved by the ethical review units of University of California, San Francisco and Kenya Medical Research Institute.

### Data analysis

We conducted descriptive analyses to examine the characteristics of the providers and their responses to the structured questions on communication and autonomy. We then examined bivariate associations between reporting on communication and autonomy and various demographics. We analyzed the qualitative data to identify themes using the approach described by Braun & Clarke [38]. We generated themes both inductively and deductively using an initial codebook based on the questions as well as codes generated from open coding 10 transcripts. This codebook was used by the rest of the team (4 coders) to code the rest of the transcripts and the codebook was continuously updated to incorporate new emerging codes from the remaining transcripts. The transcripts were divided among three coders and the lead author double coded 10 transcripts to check for consistency. New codes and identified inconsistencies were discussed to consensus by the study team. During coding, we wrote analytic and reflexive memos to capture our reactions to the data and emerging ideas. We then iteratively analyzed the codes and coded text and reviewed our memos to generate categories and identify themes. We considered both the semantic (surface) and latent (underlying) meaning of the text and focused on salience (rather than frequency) for the qualitative data [38]. Throughout the analysis, we considered how our backgrounds, training, and worldview influenced our interpretation of the results, and drew on our combined interdisciplinary insights for a broader perspective. We were unable to return transcripts to participants for comments, but the results were presented at two meetings in the county, which included some of the participants, and there was agreement that the findings reflected the situation in the county. Quantitative data were analyzed with STATA version 15 [39] and qualitative data were analyzed using Atlas.ti version 8.4 [40].

## Results

### Demographics

The characteristics of the sample shown in Table 1 have been previously described [31]. The respondents included seven clinical officers, 25 nurses and midwives, and 17 non-clinical staff (including cleaners and cooks). Thirty worked in public hospitals, 13 in health centers, and six in mission/private hospitals. Ninety percent had not had training on how to better interact with women. (See Additional file 2 for demographics by provider and facility type).

**Table 1:** Characteristics of providers

	No.	(%)
Facility type		
Govt. Hospital	30	(61.2)
Govt. Health Center	13	(26.5)
Mission Hospital	6	(12.2)
Position		
Clinical officer	7	(14.3)
Nurse/Midwife	25	(51.0)
Support staff	17	(34.6)
Female	35	(71.4)
Age		
Less than 30 years	9	(18.4)
30 to 39 years	21	(42.9)
40 or more years	19	(38.8)
Current marital status		
Single	5	(10.6)
Married	39	(83.0)
Widowed	3	(6.4)
Number of children		
0 to 2	15	(31.9)
3 or 4	21	(44.7)
5 or more	11	(23.4)
Highest education		
Less than College	17	(34.7)
College and above	32	(65.3)
From Migori County	29	(59.2)
Length of stay in Migori County		
less than 10 years	14	(28.6)
10 to 20 years	11	(22.4)
More than 20 years	24	(49.0)
Years as a provider		
0 to 5 years	18	(36.7)
6 to 10 years	13	(26.5)
More than 10 years	18	(36.7)
Number of days worked per week		
5 or fewer days	37	(77.1)
More than 5 days	11	(22.9)
Number of hours working per day		
8 or fewer hours	25	(52.1)
9 to 10 h	13	(27.1)
More than 10 h	10	(20.8)
Ever had training on how to better interact with patients		
No	44	(89.8)
Yes	5	(10.2)
Total	49	(100.0)

### Perceptions of importance of communication and autonomy

Over 80% of providers acknowledged the importance of various aspects of communication and autonomy (Table 2). For example, 86% reported that it was very important to introduce themselves to women and 79% said it was very important to call women by their names. Additionally, 84% felt it was very important to ask permission or consent before procedures and 85% said it was very important to involve women or their families in decisions about their care.

**Table 2:** Providers’ Perceptions of Importance of Communication and Women’s Autonomy

Aspect of Communication and Autonomy	Rating of importance: N (%)	Why important	Representative quotations
Providers introducing themselves to women	Not important: 2 (4.1%) Somewhat: 5 (10.2%) Very important: 42 (85.7%)	Value of women being able to identify who cared for them:	*“It is important because you may help a patient and she wants to appreciate you. Or you talked to her rudely so she will be able to tell that it is ‘so and so’ who talked to me rudely.” (NC10)* *“Sometimes when you have taken good care of a patient so she will go home happy saying that I was helped by nurse so and so”. (NC8)*
		Establishing rapport and creating an interpersonal connection with women	*“I think first is the first impression that the health provider gives the mother, so the first impression makes a great impact, so if the impression is not good then the mother might become withdrawn.” (C1)* *“Of course we [should] introduce ourselves to [patients] like when they find us in the labor ward we have to introduce ourselves to them like am so and so and am going to take you through this and this until my colleague comes.,, it is important because you create a rapport between you and the client so she will be easy for her to express her feelings...” (C20)*
Calling women by name	Not important: 0 (0%) Somewhat: 10 (20.8%) Very important: 38 (79.2%)	Recognizing the woman as an individual and expressing interest, respect and care	*“I think that if a patient tells you that she is Emily and later you call her Emily, she will feel like ‘ooh at least she can remember me, at least she has got some interest in me’.” (C4)* *“First, they [patients] are human beings, you need to respect them, and they have names. Because some people you see they will give you a report like ‘bed one’. In that ‘bed one’ there is a human being who has a name so we just need to know that they are human beings who have names” (C19)*
		Establishing rapport and creating an interpersonal connection with women	*“The patient will feel like this person knows me and so she will begin to open up.” (C18)* *“I think it is important because if you call her by her name, she feels free and she can tell you all about herself.” (C34)*
		Promoting safety	*“… also it will identify who are you really talking about so that you don’t mix patients and may be you mix up the care and now out of that you can really mess up the life of these people.” (C19)*
Explaining and consenting women before examinations and procedures	Not important: 1 (2%) Somewhat: 7 (14.3%) Very important: 41 (83.7%)	Establishing rapport and creating an interpersonal connection with women	*“It’s very important, to gain confidence or to work on the woman, you have to explain first…if at all you have not explained, then you get resistance; but if you have explained then it will go smoothly with the procedure.” (C15)* *“… when you are doing a procedure you will do it in a favorable environment, may be when you are doing a procedure and the patient is not informed or has not given you permission you will get a lot of difficulties because you will not do it the right way.” (C17)*
		Respecting women’s rights and avoiding blame or legal actions	*“Well, let me say when you look at the legal aspect of it because you may never know what can happen, pregnancy is a risk in itself so it is safe for the health care provider because in case of any legal issues then it shall have protected the health care worker and even the other thing is that, this woman will also know because if she is giving a consent of course you are going to explain, it is an informed consent, you explain what are you really going to do so this woman will also be knowing the care that she will receive” (C10)* *“That is what should be done and nowadays patients know their right and they are more informed so to be on the safer side is better you do the right thing” (C12)*
		Empowering women and their families to be stakeholders in their health	*“When you seek consent before doing any procedure the patient will be able to know that she has that power of deciding, and you will be at ease to carry out the procedure.” (C12)* *“Yes, very important to involve them in the care so that they also plan together with you and they own the care” (C1)*
Involving women and family in care	Not important: 0 (0%) Somewhat: 7 (14.6%) Very important: 41 (85.4%)	Empowering women and their families to be stakeholders in their health	*“Yes it is important because when someone brings a mother, they [relatives] would want to know whether the unborn is okay or there could be a problem and the mother needs to be referred somewhere. Once you involve them to know the mothers condition they usually become settled in the mind and thus become comfortable knowing the mother will deliver. You find some mothers make a lot of noise during labor until the relatives also get worried whether there is a problem or whether they are going to deliver normally. Once the doctor explains to them that all is okay they immediately relax” (NC18)* *“… It is important because anything can happen anytime and anywhere so if they are not aware and they are not psychologically prepared then it will be difficult to channel out your communication.” (C20)*
		Facilitating adherence, referrals and continuity of care	*“It is important because if maybe you can talk with the mother may be they will forget but if the relative is there they will remind them to take quick action before that what you are thinking may happen” (C17)* *“… when you want the continuity of it, families have to be informed so that even when the lady leaves here and gets a problem she can easily be brought back and when they go home they will remind that client whether she has taken her medicine the way she was taught..” (C4)*

On average, clinical providers were more likely than non-clinical providers to rate aspects of communication and autonomy as very important (Additional file 3). For example, while over 90% of clinical providers felt it was very important to introduce themselves, call women by name, and ask for consent; this was somewhere between 50 and 70% for non-clinical providers. There were also some differences by type of facility. For example, only 77% of the government health center staff reported that it was very important to introduce themselves compared to 93% of government hospital staff. Also, only 69% of health center staff felt it was important to ask permission or consent, compared to 87 and 100% for providers in government and mission hospitals, respectively.

Most providers were able to provide compelling reasons for why each practice was important. These reasons fall under seven themes, which are summarized below and shown in Table 2 with additional representative quotations.

### Value of women being able to identify who cared for them

Providers acknowledged that it is important to introduce themselves so that patients could identify them. For example, when they took good care of a woman, she could identify who took care of her and thank them or recommend them in the community. On the other hand, if a woman developed problems, she would know who to follow up with. Being able to identify the provider was said to increase confidentiality, such that if a woman wanted to share her problem with only that provider, she would know who to ask for when she next returned to the facility. Introducing oneself appeared to serve as an accountability mechanism for provider interactions with women, as some non-clinical providers mentioned this could motivate clinical providers to treat people well since they could potentially be identified as the ones who mistreat women.

*“When you are dealing with a patient, in case she gets better, then she is able to come back and say that maybe X helped me and others can also come and get assisted by the same doctor. And also, it will make all staff to treat patients well, because, if you don’t, then the whole community will know about you.” (NC3)*

### Recognizing the woman as an individual and expressing interest, respect and care

Many providers mentioned that calling a woman by her name recognizes her as a person, a human being, or an individual. They acknowledged that this made women feel good and respected. It also made women feel like the provider knew them, was interested in them, and cared about them. Some providers acknowledged that it was not appropriate to refer to women by bed numbers or generically as “mama,” or as “you.”

*“I think to some extent it makes a client feel like you are with the client, I mean you are interested in her, if you can know her name then you are interested in knowing about [her] and she will be open to give you everything.” (C27)*

### Establishing rapport and creating an interpersonal connection with women

Many providers acknowledged that the different practices related to communication and autonomy helped to improve the interpersonal interactions between women and providers. For example, providers mentioned that introducing themselves to the clients helped to establish rapport, make women feel comfortable, increase women’s confidence in their providers, and allow women to open up to freely discuss their problems and ask questions. Introducing themselves was linked to a positive first impression, which affected subsequent interactions. Calling women by their names was also said to create interpersonal connections, which makes women feel comfortable and free to open up to the provider. Additionally, providers noted that when women were given information and consented, it increased women’s confidence and trust in the provider, made them feel free to open up to the provider, decreased resistance to care, and promoted cooperation.

*“it [introduction] creates rapport, patient feels you are part of her, you have recognized her and this creates good relationship… This will make the client open up to you…. Because at the end, the client will have known you; and when they know you, they will have [a] comfortable feeling and she will be relaxed when you handle her.” (C30)*

### Promoting safety

Some providers mentioned that it is important to refer to women by name to make sure they accurately identified them. This was necessary to avoid mixing up their medical information and to ensure that procedures and medications were given to the correct women.

*“When you call a person by the name you will be sure it is the right patient, right treatment and also at times you might have one or two patients who are having some similar names so you will be sure whom you are attending to.” (C32)*

### Respecting women’s rights and avoiding blame or legal actions

Most respondents cited patient rights and the legal implications of not seeking consent as reasons why they always needed to seek consent. Also, giving women and their families information, seeking consent for examinations and procedures, and involving them in care were seen as protective mechanisms to ensure providers are not blamed if something goes wrong. As one provider noted: “*You cannot enter somebody’s body without her permission because if something bad happens and there was no permission you will be in jail.”* Even non-clinical providers recognized the self-protective aspects of consenting and noted that this was particularly important because women are becoming empowered.

*“Is it important to seek their permission.... to make them aware of what is going to take place, to make them feel worthy, to protect their dignity, provided for by the law and the constitution [Laugh] and to prevent the misperception of the health care worker and let the mother know what you are doing. Like when a mother, this was a case in media of a rape victim, a health worker wanted to do a vaginal examination but due to lack of informing the patient, the patient termed that as rape. The health worker was doing what is right but just because of not explaining, so to prevent such like issues.” (C1)*

### Empowering women and their families to be stakeholders in their health

Providers acknowledged that is important to check that women understand information given to them; and some reported assessing this by asking women to repeat information, giving time for questions, demonstrating, and assessing if women followed through on instructions. Providing information to women and seeking consent empowered women to be involved in their care. It helped to prepare women psychologically for procedures, made women feel they were part of the process, helped women understand they had choices, and prepared women to bear the consequences of procedures. Providers noted that family involvement prepares families for any adverse outcomes, gives families peace of mind knowing what is happening to the woman, increases family ownership in the care of the woman, and promotes family cohesion.

*“It is important in that, the patient should feel like whatever you have done to her, it is out of her choice and the patient should not feel like she was forced, and then she should be the one responsible for the consequences because you have to explain to her that if I do this, this and this might happen. She will be comfortable with the procedure and then just in case of anything she will say, I was aware and then I am ready to bear the outcome” (C14)*

### Facilitating adherence, referrals, and continuity of care

Some providers noted that family involvement helped facilitate adherence by helping remind the mother of what needs to be done during and after admission. Others noted that family involvement helped facilitate uptake of services, referrals, payments, and continuity of care. However, providers cautioned that not all information can be shared with the family and that there was a need to respect the woman’s preference for who to share her information with should be respected.

*“It is important because it will be like you are forcing if you don’t involve other relatives. Though you have explained everything, you need someone to comfort the patient and that is the relative, because when there is a referral then it is the patient with her relative who are there to make decisions.” (C15)*

### Extent of communication and women’s autonomy

Despite knowledge of the importance communication and women’s autonomy, some providers reported never engaging in certain practices or only doing so some of the time (Table 3). Providers were more likely to report negative practices for others rather than for themselves. For example, about a third (33%) reported they never introduce themselves to women, although more than half (57%) reported that other providers never introduce themselves. Over 90% of clinical providers reported that women were told the purpose of examinations, procedures, and medication most or all of the time. However, about one-third reported they did not always explain why they were doing exams or procedures and did not always explain the purpose of medicines. Over two-thirds also reported that women were not always asked for permission or consent before procedures and a similar proportion reported that providers do not always involve women and families in decision making. About one-third (38%) reported that women are never able to be in alternative positions of their choice (e.g. squatting) when giving birth. Furthermore, non-clinical providers were more likely to report that clinical staff did not perform certain practices than the clinical providers themselves, and responses suggest more effective communication and support for most aspects of women’s autonomy in the lower level facilities (health centers) than in the higher-level facilities (Additional file 3).

**Table 3:** Providers’ perceptions of extent of communication and women’s autonomy (*N* = 49)

	N (%)			
	*No, none of them*	*Yes, a few of them*	*Yes, most of them*	*Yes, all of them*
Do you think the doctors, nurses, or other health care providers introduce themselves?	28 (57.1%)	13 (26.5%)	4 (8.2%)	4 (8.2%)
Do you Introduce yourself to the women when you first see them?	16 (33.3%)	20 (41.7%)	6 (12.5%)	6 (12.5%)
Do the doctors, nurses, or other health care providers call women by their names?	6 (13.3%)	12 (14.5%)	19 (38.8%)	12 (24.5%)
Do you refer to women by their names?	5 (10.4%)	9 (18.8%)	17 (35.4%)	17 (35.4%)
Do you explain to women why you are doing examinations or procedures on them (Clinicians only)?			10 (33.3%)	20 (66.7%)
Do you explain to women why you are giving them medicines (Clinicians only)?	1 (3.3%)	2 (6.7%)	6 (20%)	21 (70%)
Do the doctors, nurses or other staff at the facility ask women permission/consent before examinations and procedures?	4 (8.2%)	4 (8.2%)	26 (53.1%)	13 (26.5%)
In your experience, are women or families given information about their care?	0 (0%)	7 (14.3%)	27 (55.1%)	15 (30.6%)
Do you feel women can ask the doctors, nurses or other staff at the facility any questions they have?	0 (0%)	8 (16.3%)	20 (40.8%)	20 (40.8%)
Do you feel women can ask you any questions they have	0 (0%)	4 (8.2%)	24 (49%)	21 (70%)
Do the doctors, nurses or other staff at the facility answer questions family have?	0 (0%)	3 (6.1%)	32 (65.3%)	14 (28.6%)
Do the doctors, nurses or other staff at the facility speak to women in a language they understand?	0 (0%)	4 (8.2%)	19 (38.8%)	26 (53.1%)
Do you feel like the doctors, nurses or other staff at the facility involve women in decisions about their care?	1 (2%)	4 (8.2%)	31 (63.3%)	13 (26.5%)
During the delivery, do you feel like women are able to be in the position of their choice?	18 (37.5%)	13 (27.1%)	12 (25%)	4 (8.3%)

### Barriers to effective communication and respect for women’s autonomy

Providers gave several reasons why they don’t always do what they know is important. These reasons group under three interrelated themes: (1) the work environment, (2) provider knowledge, intentions, and assumptions; and (3) women’s ability to demand or command effective communication and respect for their autonomy. These are presented below and summarized in Table 4.

**Table 4:** Barriers to communication and autonomy

Theme	Sub-theme	***Representative quotations***
Work environment	Perceived lack of time	*“It is the time when you have so many patients and when they ask questions you will not get time to answer them.” (C17)*
	Language barriers	*“Most of the nurses are not Luos and our women who come to this facility mainly know Luo alone so when the nurse is speaking to them in Swahili, they feel like the nurse is despising them or disrespects them. They end up nick naming them that ja Kisii [person from Kisii] is the one on duty, am not going for my clinics as she will look down upon me.” (NC12)*
	Stress and burnout	*“This mother has come with a language barrier and this person worked within the 8 h but too much work, so due to the irritability and the stress involved in it, that is when you find that instead of calling Emily Emily I call her ‘you mother’.” (C4)*
	Facility culture	*“People have set some things, we believe, it’s now a trend. I don’t know what facility you can go and find a staff introducing herself to the client like am so and so, I have never seen it.” (C8)*
Provider knowledge, intentions, and assumptions:	Inadequate provider knowledge and skill	*“Sometimes, even if the patients does have the courage to ask a question, providers may not be able to answer those questions effectively because they may not understand what the need of the relative or the mother is.” (C3)*
	Forgetfulness and unconscious behaviors	*“Yes but sometimes I usually do forget, [Laughs] you might come and you find when the line is so long … sometimes you can forget…greeting them you cannot forget, but introducing our names sometimes we do forget.” (C38)*
	Self-protection and comfort	*“Because when you introduce yourself, someone can come and say that you are the one who treated him badly and yet you were not the one.” (NC2)*
	Assumptions about women’s knowledge and expectations	*“It is the assumptions that people have that they have been here for long and they are known by most of the clients.” (C11)*
Women’s ability to demand or command effective communication and respect for their autonomy:	Women’s lack of participation	*“It is just fear, but the staffs cannot refuse to respond, like she may just but be scared from the fact that he is a doctor that is why she feels small before him and can’t ask whatever question that they have.” (NC15)*
	Women’s empowerment and provider bias	*“In most cases, you know such like patients they are too inquisitive, demanding so you will spend so much time with them when compared to the others who are not aware of what is going on.” (C32)*

#### Work environment

This theme captures institutional level factors that affect communication and women’s autonomy. Commonly providers mentioned conditions in their workplace such as workload, which led to a perceived lack of time, stress, and burnout. There was also reference to differences in background of providers and patients in some facilities, which led to miscommunication. These barriers as well as norms and other characteristics of institutions created a workplace culture that facilitated or deterred certain behaviors. We group the barriers related to work environment under four sub-themes—Perceived lack of time, language barriers, stress and burnout, and facility culture—while acknowledging the interrelationships between them.

##### Perceived lack of time

Lack of time was a common explanation for neglecting aspects of communication and autonomy. Providers felt they did not have enough time to introduce themselves, get to know women and refer to them personally, explain examinations and procedures, discuss findings, or adequately answer women and their families’ questions. One non-clinical provider stated this bluntly: *“we don’t have that time for introducing ourselves.”* This lack of time was often attributed to shortage of providers and high patient loads.

*“… sometimes maybe like now we are two in the facility, me and the Nursing Officer …..some patients are waiting for me because of the shortage…., maybe after this somebody will call me help us in the maternity, you leave this place you go there that is why sometimes we don’t even know the patients, we just tell them to remove the clothes quickly, you then quickly leave, you don’t even ask where they are coming from or how they are doing, even asking their name.” (C17)*

##### Language barriers

Language barriers were cited as another reason for not being able to provide adequate information or answer questions. Language barriers were also thought to sometimes lead women to misinterpret providers’ intentions and/or feel disrespected. Language barriers were said to be due to both providers and women not being from the community where the health facility is located and were mentioned more frequently by providers in the higher-level facilities.

*“I can also say language to some extent because you see this is a big place people come from different ethnicity and some of them come from the remote areas and they may not be able to understand the common language… and also the staffs employed here some of them may not be able to understand the language that is common here or the local language … and at times they may find themselves on duty alone.” (C1)*

##### Stress and burnout

Many of the reasons providers gave for not engaging in person-centered communication behaviors were related to stress and burnout, which was referenced directly or indirectly. Stress was often associated with fatigue, forgetfulness, impatience, irritability, and lack of attention. Factors contributing to stress and burnout included high workload, lack of essential supplies to work with, and non-work-related stress. Both perceived lack of time and language barriers, previously noted, were potential sources of stress and burnout, often interacting to exacerbate the situation.

*“…some of the staff at times maybe they are in a hurry to help the patient in the situation especially like in maternity some women they come and maybe there is that burnout you are one staff you want to do this and this, and at times you forget [to introduce yourself]” (C33)*

##### Facility culture

Facility culture affected communication and autonomy through a perceived lack of time and stress and burnout. Other direct effects of facility culture were apparent from the reasons providers gave for not introducing themselves to women. For example, a provider referenced the fact that there was no facility around where providers introduced themselves. Another clinical provider mentioned that although they were trained in school to always introduce themselves to women, it had been forgotten and was not practiced in their facilities. Non-clinical providers, in particular, who mostly learn on the job, referred to modeling the behaviors of other providers without questioning them.

*“When I started working in this facility, I found staffs not introducing themselves to the women when they first see them so we are just following what they are doing.” (NC1)*

Lack of accountability for who is responsible for communicating certain things to the women was also an issue. For example, a woman may not be told why she was given medications because there are multiple providers involved in her care and each assumed that someone else would do it.

*“…I do not know how I would put it because like here now Clinical Officers prescribes because we do not have a doctor working at this facility… maybe the pharmacy person dispenses, she/he will tell the patient this drug take two, two times a day, this other one take two three times a day but she has not been told for what purpose are they given the drugs.” (C19)*

Providers in different types of facilities also pointed to the role of facility culture. For example, non-clinical providers suggested that women to feel more comfortable in the health centers because the nurses there are “free with them”. Labor and birthing positions also appeared more prescriptive in the higher-level facilities than in the health centers.

*“We take them to the ward to give birth, only if it finds that she has not reached the bed is when she can give birth standing but if not they must be on the bed lying on their back. They are even shown how to put their legs.” (NC5)*

The physical environment also influenced the facility culture, although this was expressed less frequently in relation to poor communication and autonomy. A few providers mentioned that dirty floors in the labor room are a reason why women may not be allowed birthing positions of their choice such as squatting or birthing on the floor. Furthermore, the reason for not allowing women to birth in alternative positions was often not empathically communicated to women, which further undercut women’s autonomy in birth.

#### Provider knowledge, intentions, and assumptions

This theme captures factors at the level of the provider that affect communication and respect for women’s autonomy, and include knowledge, skills, attitudes, intentions, and assumptions. We group barriers here under four sub-themes: Inadequate provider knowledge and skill, forgetfulness and unconscious behaviors, self-protection and comfort, and assumptions about women’ knowledge and expectations.

##### Inadequate provider knowledge and skill

A few participants acknowledged that sometimes they are unable to effectively communicate due to lack of knowledge or skill. This was particularly related to understanding and answering questions. Some providers noted that sometimes they did not answer questions due to their inability to respond to sensitive questions, fear that their answers may upset or instill fear in women, or just because they were unable to understand the patient. This suggests a lack of perceptive listening skills among providers, as well as an inability to empathically communicate.

*“Lack of knowledge on the question. A good example is when a HIV positive woman and a HIV negative husband. They will ask you that how are we discordant, so questions on that line if the provider is not able to answer, either she will evade the question or find a way of dodging...” (C11)*

Inadequate knowledge was apparent in non-clinical providers response to certain questions. For example, one non-clinical provider’s response to the question on whether providers explained the purpose of examinations and procedures indicated that they were interpreting this to be equivalent to giving instructions. Some also believed that women are supposed to do whatever the doctor tells them, so they did not think women should have a preference in their birthing positions. Some non-clinical providers also tended to overestimate the knowledge of clinical providers by stating they “know everything” and can always answer questions from women.

*“she is to take the position she is told by the doctor; you cannot allow her to take her own position.” (NC7)*

##### Forgetfulness and unconscious behaviors verses self-protection and comfort

Sometimes provider behaviors were subconscious. For example, many providers said they sometimes forgot to do certain things such as introducing themselves or explaining procedures. This was often because it was not something that was frequently done in their facilities. Forgetfulness was also attributed to stress from the high workload or in the case of emergencies.

*“…it is just something that is hard for one to remember that I have to introduce myself. At times we tend to forget… I do not know whether it is a habit or assumption that we just don’t.” (C7)*

Other times, it appeared that providers consciously decided to behave in certain ways to protect themselves. For example, some providers mentioned that sometimes they don’t introduce themselves because they don’t want women to know their names. These providers fear that they may be mistakenly identified as someone who mistreated a patient or that they will be identified (and held accountable) if they actually do mistreat a patient. This concern about being identified when they mistreat a woman was mostly expressed by the non-clinical providers. However, non-clinical providers referred to a similar rationale for clinical providers, and some even suggested that clinical providers might be less likely to mistreat women if women knew them by name, as they would be able to identify the person who mistreated them.

*“You may deal with a patient in a bad way, so if you introduce yourself to her then she will know you more and tell others about you. So it’s better not to introduce yourself to them… or you talked to her rudely so she will be able to tell that it is so and so who talked to me rudely. (NC10)*

Also, some were concerned about women calling them by their names outside of the facility. In addition, some felt clinical providers might not want to introduce themselves because women will go and talk about them in the community or they will refer to them by their names instead of by their title such as “doctor.”

*“Some think that when they introduce themselves, then the women will go and start talking about them in the community. Maybe the doctor or nurse did something bad to the patient so if they say their names, the patient will go and tell others about her outside. So that is why they don’t introduce themselves to the patients” (NC3)*

Provider protection and comfort both negatively and positively affected communication and autonomy. For example, consenting was thought of as a means of protecting providers from litigation, which motivated some to seek consent for procedures. On the other hand, it appeared that women were sometimes prevented from giving birth in their preferred positions because those positions were not comfortable for the provider.

*“Because some of them, they prefer the squatting position and may be the staff who is there may have it difficult to kneel down… At times especially that position is also uncomfortable for the health worker.” (C4)*

##### Assumptions about women’ knowledge and expectations

Providers sometimes attributed their behaviors to their assumptions of women’s knowledge and expectations, which may or not always be correct. For example, many providers said they don’t usually introduce themselves because they assume the women already know them or ought to know them, as most of them came from the same community. Some also mentioned that women will know who they are by their uniforms or by their name tags. One provider said that once you are in a uniform or lab coat, you don’t have to introduce yourself. Others, however, admitted that although women can know they are health providers by virtue of them being in the community or by their uniforms, they don’t necessarily know their names.

*“No, that is a weakness I have, I normally assume that they know me, but when I am doing health talks I introduce myself…It is a preconceived idea that they know us.” (C4)*

Some also felt that some women did not expect them to introduce themselves or may not be interested in them introducing themselves because of their condition. Non-clinical providers were more likely to have this opinion.

*“The patient has come and is sick, she is not interested in knowing your name but to get treated. Some patients come while in bad mental condition, how can you introduce yourself to such a person, all she is busy with is to get life back.” (NC13)*

Some providers admitted to not providing women with enough information about their care or adequately obtaining their consent because of assumptions that: women already knew what was happening (although providers often acknowledged that this was not always true), may not understand what they were told, or may be frightened by the information.

*“Most of the times they just examine them as they assume that the women know why they are examining them. But some young girls do not know and the mothers who are used to giving birth at home do not know too and so they should always ask for permission from all of them so that they get to know why the nurses are doing anything in them.” (NC1)*

In addition, some providers said they assumed that coming to the facility meant a woman had given her consent for *all* care given at the facility and they, therefore, did not need to ask for permission or consent to do something. These misconceptions were higher among non-clinical providers, though they were also expressed by some clinical providers.

*“when a mother comes voluntarily to deliver, that is a sign that she has given the doctor permission to treat and assist her in whichever way. So I do not see whether there is need again for a doctor to ask permission from her before he does a procedure or examination.” (NC11)*

#### Women’s ability to demand or command effective communication and respect for their autonomy

This theme captures factors based on women’s characteristics that contribute to differences in communication and respect for women’s autonomy. There are two sub-themes related to this main theme: Women’s lack of participation and women’s empowerment which is influenced by provider bias.

##### Women’s lack of participation

Providers sometimes attributed poor communication to a lack of participation from the women. For example, some providers reported that some women do not ask questions because of fear, shame, and feeling inferior to providers. Similarly, some providers ascribed lack of autonomy to women’s passivity. In particular, women not birthing in their preferred positions was attributed to them not asking.

*“This facility is like in a community, it is not in the Urban set up, so will not really hear any woman who will ask you sister I want to deliver while squatting, they will not ask, they will just assume the position that they will know {laughter…} no one will ask you I want to lie like this, I want to give birth squatting if they are not asking then of course even us {laughter…} we cannot push them.” (C19)*

Responses from non-clinical providers however suggested that birthing in alternative position was not often an option for women. Additionally, it was also evident that even when women asked, they might be denied based on the type of positions they preferred due to provider discomfort or the state of the labor ward. In addition, provider assumptions about why they were requesting that position were often biased. An example of this includes a provider assuming that a woman wants to deliver on the floor because she perceived the tiles to be cleaner than the delivery bed (because they do not have tiles in their homes).

*“No, they are forced to lie on their backs… they are told to lie on their backs and hold their legs….” (NC13)*

*“… there are some who want to deliver down on the floor, that one we do not allow, you must deliver on the couch. When they come they tend to see this tiles to be cleaner than the couch and we don’t allow that,” (C18)*

Women’s interactions with providers were said to be influenced by provider characteristics. It was said that women may be less likely to communicate effectively with male providers or younger providers because of fear or shyness. Non-clinical providers were more likely to mention that females may be uncomfortable asking male providers questions because of fear and perceived inferiority. Some non-clinical providers reported facilitating communication between women and clinical providers when women did not feel that they were able to go directly to clinical providers with their questions.

*“Some fear doctors for example if the doctor is a male so they come to me to ask me, then I go back to the doctor together with her and we ask and we both get the responses and I also learn from there… like when she has an itchy private part, they cannot explain to the male nurse so they will just go back home with the problem.”(NC12)*

Some providers, however, acknowledged that a woman’s behavior might be a response to provider behavior. A woman’s comfort or confidence in asking questions was affected by the way in which she was treated by her provider. For example, some women feared to ask questions when the provider was not friendly or did not receive them well.

*“I think the first thing is how this woman has been received and who has received this woman, has she or he built a relationship with this woman, has she/he created that friendly environment because if the approach is bad from the beginning then this woman will not be open” (C19)*

##### Women’s empowerment and provider bias

There was a bias towards providing more information and respecting the autonomy of women that providers believed were well informed, of a higher status, and/or more empowered. Providers noted that it was safe to do the right thing when women are well informed because they know their rights. Also, well informed women were said to ask more questions and to be more demanding, resulting in providers spending more time with them. Women’s companions also influenced communication and autonomy. When women had someone to advocate for them, they were more likely to receive information about their care.

*“nowadays patients know their right and they are more informed so to be on the safer side is better you do the right thing” (C12)*

### Facilitators of effective communication and respect for women’s autonomy

Because of the way the guide for the interview was set up, the discussion on communication and autonomy mostly focused on barriers, hence our focus on that in this manuscript. However, when a general question was asked on how to improve patient provider interactions, providers usually mentioned addressing the issues they had already discussed. This implies that addressing the barriers discussed above will facilitate effective communication and respect for women’s autonomy. Of note many providers also mentioned training as a means to improve patient-provider interactions. This included training on how to talk to patients to build trust and confidence and how to talk to hostile and uncooperative patients. Many also mentioned training on stress and a few mentioned trainings on discrimination.

*“I would like to train on how to relate with patients and how to manage stress at work” (NC3)*

*“[training on] how to handle patients who are hostile and who cannot cooperate and … how can you build confidence in patients so that they become free and open up” (C21)*

## Discussion

This paper is among the first in sub-Saharan Africa to explore in detail providers’ perceptions on various aspects of communication and autonomy during childbirth. It is a mixed-methods study with both clinical and non-clinical providers working in different levels of facilities in a rural county in Western Kenya. We find that despite a relatively strong awareness of the importance and benefits of effective communication and respect for women’s autonomy, the associated recommended behaviors are not consistently practiced by providers. The barriers to effective communication and autonomy are related to the work environment; provider knowledge, intentions, and assumptions; and women’s ability to demand or command effective communication and respect for their autonomy. Addressing these barriers are critical to facilitating effective communication and respect for women’s autonomy.

The finding that communication and autonomy is sub-optimal in many facilities is consistent with findings from other studies with providers [21]. Studies with women however paint a more dire situation. For example, in a study with women in the same setting, 77% reported that providers never introduced themselves to them and 70% were never allowed to deliver in a position of their choice [41]. Also 28% were never told the purpose of examinations and procedures and 36% were never asked permission or consent before examinations and procedures. These numbers imply poorer communication and autonomy than what was reported by the providers. However, given that provider responses regarding their own behaviors are more prone to social desirability bias, it could be assumed that providers overestimated the extent of communication and autonomy in their facilities. This incongruence has been shown in other studies [42].

This paper contributes to understanding of the “know-do” gap in PCMC. Although most providers recognized the importance of various practices related to communication and autonomy, they did not always engage in these practices (and some never did). A key reason for the “know-do” gap is the work environment. The sub-themes under work environment such as perceived lack of time, stress and burnout, and facility culture are known barriers to effective communication and other dimensions of quality care [19, 43]. Staff shortage and the resulting high patient load leads to stress among providers, as well as a perceived lack of time, which caused them to forget or ignore recommended practices. Persistent high stress leads to burnout which results in poor interactions with women—with implications for lower levels of patient safety and quality of care in general [44, 45]. Also, when providers feel they have limited time to attend to all their patients, they prioritize what they deem is most important and efficient, which is usually providing clinical care and dispensing medications. With many providers not engaging in good communication practices, it becomes the norm and accepted culture in the facilities, further perpetuating poor communication and a lack of patient autonomy in facilities.

The facility culture influences what providers remember to do, hence the providers’ responses which suggest that poor communication is sometimes unintentional. There were also times when providers consciously avoided or engaged in certain behaviors in order to protect themselves. The fear of legal action had the positive effect of promoting communication and patient autonomy. However, this implies that providers may be less likely to provide information or respect the autonomy of women that they believe may not be able to seek legal redress. The differences by type of facility also support the role of facility culture in the “know-do” gap. There seemed to be a sense of community at lower level facilities that improved patient-provider interactions in those facilities. The differences in communication and autonomy by facility type are consistent with findings from interviews with women suggesting lower PCMC in higher level facilities [26, 41].

These findings do not mean provider knowledge is not important, but that knowledge alone is not sufficient. Inadequate knowledge was a bigger issue among non-clinical providers, who were less likely to rate the practices as very important. All non-clinical providers reported they had never had a training on how to interact with patients—despite the evidence that they directly interacted with patients and played a role in shaping women’s maternity experiences in that setting [16, 30]. Non-clinical providers, therefore, essentially learned how to interact with patients by watching the clinical providers, whose communication skills they sometimes overestimated. Many of the clinical providers had, however, never undergone training on how to interact with patients themselves; and some admitted that poor communication was sometimes due to inadequate knowledge and skills, especially when it involved sensitive issues.

Finally, a woman’s inability to ask for what she wants or command quality care by virtue of her status further contributes to poor communication and autonomy. A woman’s ability to express herself is, however, usually in response to specific provider behaviors, patient-provider power dynamics, as well as by the facility’s culture. Inadequate knowledge and disempowerment coupled with providers’ attitudes and biases towards these women—both consciously and unconsciously—disincentivizes women to participate in their own care. This further reinforces poor communication, as well as lack of respect for their autonomy. Prior studies, including studies in the same county, have shown that women of low social status (poor, illiterate, and unemployed) have poorer antenatal and maternity experiences in health facilities [26, 41, 46].

### Strengths and limitations

The main limitation of this study is the effect of social desirability bias, as providers were asked to report on their own behaviors. We did not have the resources to systematically conduct observations. Thus, the practice of providers on the various aspects of communication and autonomy are likely overestimated. Also, the focus on salience rather than frequency in the qualitative analysis implies that the themes do not necessarily reflect the frequency of occurrence of issues related to that theme. The quantitative data addresses this issue but is also limited because of the relatively small sample size. Selection bias is also a potential bias, as the data represents the views of providers from high volume birthing facilities, which are not be representative of all facilities in the county. In particular, the issues of workload may be over-represented from these busier facilities. Also, given that the data was collected in one county, the results may not be generalizable beyond the study county.

Nonetheless, this study has several strengths. It is among the few studies to examine the issue of communication and autonomy in detail from the perspective of both clinical and non-clinical providers. Including non-clinical providers may have helped to reduce social desirability bias, as non-clinical providers were more of participant observers in clinical care (this was not always true from other data in the study, as some non-clinical providers reported being involved in assisting with births in some facilities), and not knowing what the “right answer” is may have led to less socially desirable answers from non-clinical providers. Also, including providers from different levels and types of facilities allowed exploration of factors that might differ across them. Finally, using a mixed methods design allowed for estimation of extent of various practices as well as in-depth understanding of perceptions of importance and barriers.

### Implications for policy and practice

This study highlights that knowledge of the importance of effective communication and autonomy is essential, but it is not sufficient for engaging in person-centered behaviors. This implies that interventions need to go beyond didactic sessions on importance of communication and autonomy and patient rights, to ones that provide them with the tools to effectively bridge the know-do gap. Trainings that give providers the opportunity to practice person-centered communication in the context of providing clinical care could help them develop the skills to communicate effectively and respect women’s autonomy even under time pressure [47]. Trainings also need to include content that address self-centered intentions and wrong assumptions, stress management, and explicit and implicit biases that lead to discrimination. Importantly, these trainings should include *all* providers in a facility, including non-clinical providers. Having all providers in a facility undergo a training will help provide peer reinforcement and accountability, which can catalyze sustainable change in the culture of the facility.

Training programs alone will not be enough. Interventions need to address the various drivers of poor communication and autonomy highlighted by the providers. This should include improvements to the work environment and facility culture to enable providers to practice what they know. Strengthening health systems to address workload, stress and burnout, and putting in place measures to reduce language barriers would facilitate effective communication and respect for women’s autonomy. Interventions also need to be tailored to the needs of providers in different levels of facilities. In addition, educating women on their rights would empower them to be better co-actors in their care. Women’s empowerment initiatives beyond the health facility would contribute to their receipt of high-quality care.

Although this paper focuses on communication and autonomy, it is important to note the link between communication and autonomy and other dimensions of PCMC. For example, in other data from this study, providers highlighted that disrespect and abuse of women is often the result of women being perceived as uncooperative and difficult [32]. In this study, however, providers acknowledged how various aspects of communication and autonomy can increase the likelihood that women will be cooperative. Helping providers see the link between effective communication and difficult situations, and helping them develop their skills for effective communication in difficult situations could help reduce other forms of poor PCMC. In addition, getting providers to practice certain behaviors such as consistently introducing themselves could directly improve women’s experiences, as well as serve indirectly as an accountability mechanism against mistreatment.

## Conclusions

This study is among the few in low-resource settings on providers’ perspectives of communication and autonomy in their facilities that includes both clinical and support staff, as well as providers at different levels of facilities—including private facilities. Our findings support evidence of poor communication and autonomy found in prior studies from interviews with postpartum women. Despite an awareness of the importance of various aspects of communication and autonomy, providers report various barriers to practicing effective communication and autonomy, which leads to a “know-do” gap. Interventions should therefore reinforce what providers know and accept, teach what they don’t know, and address the barriers to applying their knowledge. Such interventions should go beyond training to address systemic factors such as increased workforce and accountability mechanisms, as well as the empowerment of women. In addition, there is a need for a cultural shift towards a more person-centered culture. This will help ensure sustainable efforts to improve person-centered/respectful maternity care within the broader framework of providing high quality universal health care.

## Supplementary information

**Additional file 1.** Consolidated criteria for reporting qualitative studies (COREQ): 32-item checklist.

**Additional file 2.** Characteristics of providers by provider type and facility type.

**Additional file 3. **Providers’ perceptions of extent of communication and autonomy by provider type and facility type.

## Data Availability

The data analyzed for the manuscript are available from the corresponding author on reasonable request.

## Abbreviations

PCMC: Person-centered maternity care
WHO: World Health Organization

## References

[1] Miller S, Abalos E, Chamillard M, Ciapponi A, Colaci D, Comandé D, et al. Beyond too little, too late and too much, too soon: a pathway towards evidence-based, respectful maternity care worldwide. Lancet. 2016. 10.1016/S0140-6736(16)31472-6. 10.1016/S0140-6736(16)31472-6 27642019

[2] Koblinsky M, Moyer CA, Calvert C, Campbell J, Campbell OMR, Feigl AB, et al. Quality maternity care for every woman, everywhere: a call to action. Lancet. 2016. 10.1016/S0140-6736(16)31333-2. 10.1016/S0140-6736(16)31333-2 27642018

[3] Kruk ME, Gage AD, Joseph NT, Danaei G, García-Saisó S, Salomon JA. Mortality due to low-quality health systems in the universal health coverage era: a systematic analysis of amenable deaths in 137 countries. Lancet. 2018. 10.1016/S0140-6736(18)31668-4. 10.1016/S0140-6736(18)31668-4 PMC6238021 30195398

[4] Tunçalp Ӧ, Were W, MacLennan C, Oladapo O, Gülmezoglu A, Bahl R (2015). Quality of care for pregnant women and newborns—the WHO vision. BJOG Int J Obstet Gynaecol.

[5] World Health Organisation (2016). WHO recommendations on antenatal care for a positive pregnancy experience.

[6] Donabedian A (1988). The quality of care. How can it be assessed?. JAMA J Am Med Assoc.

[7] Institute of Medicine (2001). Crossing the quality chasm: a new health system for the 21st century.

[8] World Health Organization (2006). Quality of care : a process for making strategic choices in health systems.

[9] Rice N, Robone S, Smith PC. The measurement and comparison of health system responsiveness: Health, Econometrics and Data Group (HEDG) Working Paper. HEDG, c/o Department of Economics, University of York; 2008. http://ideas.repec.org/p/yor/hectdg/08-05.html. Accessed 20 June 2013.

[10] Robone S, Rice N, Smith PC (2011). Health systems’ responsiveness and its characteristics: a cross-country comparative analysis. Health Serv Res.

[11] Stewart MA (1995). Effective physician-patient communication and health outcomes: a review. CMAJ Can Med Assoc J J Assoc Medicale Can.

[12] Pinto RZ, Ferreira ML, Oliveira VC, Franco MR, Adams R, Maher CG (2012). Patient-centred communication is associated with positive therapeutic alliance: a systematic review. J Phys.

[13] Doyle C, Lennox L, Bell D. A systematic review of evidence on the links between patient experience and clinical safety and effectiveness. BMJ Open. 2013;3. 10.1136/bmjopen-2012-001570. 10.1136/bmjopen-2012-001570 PMC3549241 23293244

[14] Oliveira VC, Refshauge KM, Ferreira ML, Pinto RZ, Beckenkamp PR, Negrao Filho RF (2012). Communication that values patient autonomy is associated with satisfaction with care: a systematic review. J Phys.

[15] Stewart M, Brown JB, Donner A, McWhinney IR, Oates J, Weston WW (2000). The impact of patient-centered care on outcomes. J Fam Pract.

[16] Afulani PA, Kirumbi L, Lyndon A. What makes or mars the facility-based childbirth experience: thematic analysis of women’s childbirth experiences in western Kenya. Reprod Health. 2017;14. 10.1186/s12978-017-0446-7. 10.1186/s12978-017-0446-7 PMC5747138 29284490

[17] Downe S, Finlayson K, Oladapo O, Bonet M, Gülmezoglu AM (2018). What matters to women during childbirth: a systematic qualitative review. PLoS One.

[18] Shakibazadeh E, Namadian M, Bohren MA, Vogel JP, Rashidian A, Nogueira Pileggi V (2018). Respectful care during childbirth in health facilities globally: a qualitative evidence synthesis. BJOG Int J Obstet Gynaecol.

[19] World Health Organization. WHO recommendations: intrapartum care for a positive childbirth experience: WHO; 2018. http://www.who.int/reproductivehealth/publications/intrapartum-care-guidelines/en/. Accessed 28 Feb 2018. 30070803

[20] Abuya T, Warren CE, Miller N, Njuki R, Ndwiga C, Maranga A, et al. Exploring the prevalence of disrespect and abuse during childbirth in Kenya. PLoS One. 2015;10. 10.1371/journal.pone.0123606. 10.1371/journal.pone.0123606 PMC4401776 25884566

[21] Asefa A, Bekele D, Morgan A, Kermode M (2018). Service providers’ experiences of disrespectful and abusive behavior towards women during facility based childbirth in Addis Ababa, Ethiopia. Reprod Health.

[22] Asefa A, Bekele D. Status of respectful and non-abusive care during facility-based childbirth in a hospital and health centers in Addis Ababa, Ethiopia. Reprod Health. 2015;12. 10.1186/s12978-015-0024-9. 10.1186/s12978-015-0024-9 PMC4403719 25890317

[23] Bohren MA, Vogel JP, Hunter EC, Lutsiv O, Makh SK, Souza JP, et al. The mistreatment of women during childbirth in health facilities globally: a mixed-methods systematic review. PLoS Med. 2015;12. 10.1371/journal.pmed.1001847. 10.1371/journal.pmed.1001847 PMC4488322 26126110

[24] Mannava P, Durrant K, Fisher J, Chersich M, Luchters S (2015). Attitudes and behaviours of maternal health care providers in interactions with clients: a systematic review. Glob Health.

[25] Okafor II, Ugwu EO, Obi SN (2015). Disrespect and abuse during facility-based childbirth in a low-income country. Int J Gynaecol Obstet Off Organ Int Fed Gynaecol Obstet.

[26] Afulani PA, Phillips B, Aborigo RA, Moyer CA (2019). Person-centred maternity care in low-income and middle-income countries: analysis of data from Kenya, Ghana, and India. Lancet Glob Health.

[27] Chang Y-S, Coxon K, Portela AG, Furuta M, Bick D (2018). Interventions to support effective communication between maternity care staff and women in labour: a mixed-methods systematic review. Midwifery..

[28] Leonard KL, Masatu MC (2010). Professionalism and the know-do gap: exploring intrinsic motivation among health workers in Tanzania. Health Econ.

[29] Mohanan M, Vera-Hernández M, Das V, Giardili S, Goldhaber-Fiebert JD, Rabin TL (2015). The know-do gap in quality of health care for childhood diarrhea and pneumonia in rural India. JAMA Pediatr.

[30] Golub G, Sudhinaraset M, Giessler K, Dunlop-Korsness K, Stone A (2020). The extended role of health facility cleaners in maternity care in Kenya. Int Perspect Sex Reprod Health.

[31] Afulani P, Kusi C, Kirumbi L, Walker D (2018). Companionship during facility-based childbirth: results from a mixed-methods study with recently delivered women and providers in Kenya. BMC Pregnancy Childbirth.

[32] Afulani PA, Kelly AM, Buback L, Asunka J, Kirumbi L, Lyndon A. Providers’ perceptions of disrespect and abuse during childbirth: a mixed-methods study in Kenya. Health Policy Plan. 2020. 10.1093/heapol/czaa009. 10.1093/heapol/czaa009 PMC7225569 32154878

[33] MCD (2016). Department of Health Services.

[34] Health Policy Project (2015). Kenya county health fact sheets.

[35] Kenya National Bureau of Statistics, Ministry of Health, National AIDS Control Council, Kenya Medical Research Institute, National Council for Population and Development, Nairobi, Kenya, and The DHS Program, ICF International, Rockville, Maryland, USA (2015). The DHS program - Kenya: DHS, 2014 - final report (English).

[36] Afulani PA, Diamond-Smith N, Golub G, Sudhinaraset M. Development of a tool to measure person-centered maternity care in developing settings: validation in a rural and urban Kenyan population. Reprod Health. 2017;14. 10.1186/s12978-017-0381-7. 10.1186/s12978-017-0381-7 PMC5610540 28938885

[37] Harris PA, Taylor R, Thielke R, Payne J, Gonzalez N, Conde JG (2009). Research electronic data capture (REDCap) - a metadata-driven methodology and workflow process for providing translational research informatics support. J Biomed Inform.

[38] Braun V, Clarke V (2006). Using thematic analysis in psychology. Qual Res Psychol.

[39] StataCorp (2017). Stata statistical software: release 15.

[40] ATLAS.ti (2016). ATLAS.ti: the qualitative data analysis & research software. atlas.ti.

[41] Afulani PA, Sayi TS, Montagu D (2018). Predictors of person-centered maternity care: the role of socioeconomic status, empowerment, and facility type. BMC Health Serv Res.

[42] Sudhinaraset M, Giessler K, Golub G, Afulani P (2019). Providers and women’s perspectives on person-centered maternity care: a mixed methods study in Kenya. Int J Equity Health.

[43] Bashour HN, Kanaan M, Kharouf MH, Abdulsalam AA, Tabbaa MA, Cheikha SA. The effect of training doctors in communication skills on women’s satisfaction with doctor-woman relationship during labour and delivery: a stepped wedge cluster randomised trial in Damascus. BMJ Open. 2013;3. 10.1136/bmjopen-2013-002674 PMC3752047 23945729

[44] Maslach C, Leiter MP (2016). Understanding the burnout experience: recent research and its implications for psychiatry. World Psychiatry Off J World Psychiatr Assoc WPA.

[45] Maslach C, Schaufeli WB, Leiter MP (2001). Job burnout. Annu Rev Psychol.

[46] Afulani PA (2015). Rural/urban and socioeconomic differentials in quality of antenatal care in Ghana. PLoS One.

[47] Afulani PA, Aborigo RA, Walker D, Moyer CA, Cohen S, Williams J (2019). Can an integrated obstetric emergency simulation training improve respectful maternity care? Results from a pilot study in Ghana. Birth Berkeley Calif.

