# Supplementary material for: Providers’ perceptions of communication and women’s autonomy during childbirth: a mixed methods study in Kenya
Source: Reprod Health. 2020 Jun 3;17:85. doi: 10.1186/s12978-020-0909-0 (PMC7268432; doi:10.1186/s12978-020-0909-0)
Supplement: Supplementary file 1 — Additional file 1. Consolidated criteria for reporting qualitative studies (COREQ): 32-item checklist. [file 12978_2020_909_MOESM1_ESM.pdf]

Additional file 1: Consolidated criteria for reporting qualitative studies (COREQ): 32-item checklist

No Item	Guide questions/description	Response	manuscript section
Domain 1: Research team and reflexivity			
Personal Characteristics			
1. Interviewer/facilitator	Which author/s conducted the interview or focus group?	First author trained research assistants to conduct interviews	Methods: paragraphs 2
2. Credentials	What were the researcher's credentials? E.g. PhD, MD	Lead Author: MBCHB, MPH, PhD. Other researchers with MPH; MSc; MD (OBGYN), MPH; MBChB, MMed (OBGYN); PhD, RNC	Cover page and Methods paragraphs 2
3. Occupation	What was their occupation at the time of the study?	Lead author was Postdoctoral fellow during data collection and an assistant professor during analysis. Second author is a research analyst. Third author was a master's in global health student and subsequently a medical student. Fourth author is senior research scientist, and last two authors are professors	Methods paragraphs 2 and Funding
4. Gender	Was the researcher male or female?	5 authors and research assistants are female; one author is male	Methods paragraphs 2

5. Experience and training	What experience or training did the researcher have?	Lead author has qualitative and mixed methods training and research experience; last author is a qualitative researcher who teaches qualitative research methods. Other authors have various experiencse in global MCH research	Methods paragraphs 2
Relationship with participants			
6. Relationship established	Was a relationship established prior to study commencement?	No, except contact extablished by research assistants	Methods paragraphs 2
7. Participant knowledge of the interviewer	What did the participants know about the researcher? e.g. personal goals, reasons for doing the research	Participants told purpose of the research. They did not know the researcher	Methods paragraphs 2
8. Interviewer characteristics	What characteristics were reported about the interviewer/facilitator? e.g. Bias, assumptions, reasons and interests in the research topic	Training and role in study	Methods: paragraphs 2
Domain 2: study design			
Theoretical framework			
9. Methodological orientation and Theory	What methodological orientation was stated to underpin the study? e.g. grounded theory, discourse analysis, ethnography, phenomenology, content analysis	Thematic in a mixed methods study. We applied applied Braun and Clark's approach to thematic analysis	Methods: data analysis paragraphs 1
Participant selection			
10. Sampling	How were participants selected? e.g. purposive, convenience, consecutive, snowball	Purposive	Methods: paragraph 2
11. Method of approach	How were participants approached? e.g. face-to-face, telephone, mail, email	Face to face	Methods: paragraph 2
12. Sample size	How many participants were in the study?	49	Methods: paragraph 2
13. Non-participation	How many people refused to participate or dropped out? Reasons?	None	Methods: paragraph 2

Setting			
14. Setting of data collection	Where was the data collected? e.g. home, clinic, workplace	Health facilities	Methods: paragraph 2
15. Presence of non-participants	Was anyone else present besides the participants and researchers?	No	Methods: paragraph 2
16. Description of sample	What are the important characteristics of the sample? e.g. demographic data, date	Interview period and demographic information provided	Methods: paragraph 2 and Table 1
Data collection			
17. Interview guide	Were questions, prompts, guides provided by the authors? Was it pilot tested?	Yes	Methods: paragraph 2
18. Repeat interviews	Were repeat interviews carried out? If yes, how many?	No	Methods: paragraph 2
19. Audio/visual recording	Did the research use audio or visual recording to collect the data?	Yes; audio	Methods: paragraph 3
20. Field notes	Were field notes made during and/or after the interview or focus group?	Yes, but they were not coded for the manuscript	N/A
21. Duration	What was the duration of the interviews or focus group?	1 hour	Methods: paragraph 3
22. Data saturation	Was data saturation discussed?	Yes	methods: data analysis paragraphs 1
23. Transcripts returned	Were transcripts returned to participants for comment and/or correction?	No	methods: data analysis paragraphs 1
Domain 3: analysis and findings			
Data analysis			
24. Number of data coders	How many data coders coded the data?	4	methods: data analysis paragraphs 1
25. Description of the coding tree	Did authors provide a description of the coding tree?	Yes	methods: data analysis paragraphs 1 and tables 2 and 4
26. Derivation of themes	Were themes identified in advance or derived from the data?	Derived from data	methods: data analysis paragraphs 1
27. Software	What software, if applicable, was used to manage the data?	Atlas ti	methods: data analysis paragraphs 1

28. Participant checking	Did participants provide feedback on the findings?	No, but the findings were presented at two different sessions to providers in the county, and there was general agreement that the findings reflected the situation in the county	methods: data analysis paragraphs 1
Reporting			
29. Quotations presented	Were participant quotations presented to illustrate the themes / findings? Was each quotation identified? e.g. participant number	Yes	Results: text and tables 2 and 4
30. Data and findings consistent	Was there consistency between the data presented and the findings?	Yes	Results: text and tables 2 and 4
31. Clarity of major themes	Were major themes clearly presented in the findings?	Yes	Results: text and tables 2 and 4
32. Clarity of minor themes	Is there a description of diverse cases or discussion of minor themes?	Yes; results	Results: Differences between clinical and non-clinical providers and providers from different facilities integrated in results
