# Supplementary material for: Providers’ perceptions of communication and women’s autonomy during childbirth: a mixed methods study in Kenya
Source: Reprod Health. 2020 Jun 3;17:85. doi: 10.1186/s12978-020-0909-0 (PMC7268432; doi:10.1186/s12978-020-0909-0)
Supplement: Supplementary file 2 — Additional file 2. Characteristics of providers by provider type and facility type. [file 12978_2020_909_MOESM2_ESM.docx]

| **Additional file 2: Characteristics of providers by provider type and facility type** | | | | | | | | | | | | | | | | | |
| --- | --- | --- | --- | --- | --- | --- | --- | --- | --- | --- | --- | --- | --- | --- | --- | --- | --- |
|  |  |  |  | Provider type | | | | |  | Facility type | | | | | | | |
|  | *Total* | |  | *Clinical staff* | |  | *Support staff* | |  | *Govt. Hospital* | |  | *Govt. Health Center* | |  | *Mission Hospital* | |
|  | No. | % |  | No. | % |  | No. | % |  | No. | % |  | No. | % |  | No. | % |
| Facility type |  |  |  |  |  |  |  |  |  |  |  |  |  |  |  |  |  |
| Govt. Hospital | 30 | 61.2 |  | 19 | 59.4 |  | 11 | 64.7 |  | 30 | 100 |  | 0 | 0 |  | 0 | 0 |
| Govt. Health Center | 13 | 26.5 |  | 9 | 28.1 |  | 4 | 23.5 |  | 0 | 0 |  | 13 | 100 |  | 0 | 0 |
| Mission Hospital | 6 | 12.2 |  | 4 | 12.5 |  | 2 | 11.8 |  | 0 | 0 |  | 0 | 0 |  | 6 | 100 |
| Position |  |  |  |  |  |  |  |  |  |  |  |  |  |  |  |  |  |
| Clinical officer | 7 | 14.3 |  | 7 | 21.9 |  | 0 | 0 |  | 2 | 6.7 |  | 4 | 30.8 |  | 1 | 16.7 |
| Nurse/Midwife | 25 | 51 |  | 25 | 78.1 |  | 0 | 0 |  | 17 | 56.7 |  | 5 | 38.5 |  | 3 | 50 |
| Support staff | 17 | 34.6 |  | 0 | 0 |  | 17 | 100 |  | 11 | 36.6 |  | 4 | 30.8 |  | 2 | 33.4 |
| Sex |  |  |  |  |  |  |  |  |  |  |  |  |  |  |  |  |  |
| Male | 14 | 28.6 |  | 12 | 37.5 |  | 2 | 11.8 |  | 6 | 20 |  | 6 | 46.2 |  | 2 | 33.3 |
| Female | 35 | 71.4 |  | 20 | 62.5 |  | 15 | 88.2 |  | 24 | 80 |  | 7 | 53.8 |  | 4 | 66.7 |
| Age |  |  |  |  |  |  |  |  |  |  |  |  |  |  |  |  |  |
| Less than 30 years | 9 | 18.4 |  | 7 | 21.9 |  | 2 | 11.8 |  | 2 | 6.7 |  | 4 | 30.8 |  | 3 | 50 |
| 30 to 39 years | 21 | 42.9 |  | 15 | 46.9 |  | 6 | 35.3 |  | 13 | 43.3 |  | 7 | 53.8 |  | 1 | 16.7 |
| 40 or more years | 19 | 38.8 |  | 10 | 31.2 |  | 9 | 52.9 |  | 15 | 50 |  | 2 | 15.4 |  | 2 | 33.3 |
| Current marital status |  |  |  |  |  |  |  |  |  |  |  |  |  |  |  |  |  |
| Single | 5 | 10.6 |  | 5 | 16.1 |  | 0 | 0 |  | 2 | 6.9 |  | 1 | 8.3 |  | 2 | 33.3 |
| Married | 39 | 83 |  | 25 | 80.6 |  | 14 | 87.5 |  | 24 | 82.8 |  | 11 | 91.7 |  | 4 | 66.7 |
| Widowed | 3 | 6.4 |  | 1 | 3.2 |  | 2 | 12.5 |  | 3 | 10.3 |  | 0 | 0 |  | 0 | 0 |
| Number of children |  |  |  |  |  |  |  |  |  |  |  |  |  |  |  |  |  |
| 0 to 2 | 15 | 31.9 |  | 13 | 43.3 |  | 2 | 11.8 |  | 5 | 16.7 |  | 7 | 58.3 |  | 3 | 60 |
| 3 or 4 | 21 | 44.7 |  | 14 | 46.7 |  | 7 | 41.2 |  | 16 | 53.3 |  | 5 | 41.7 |  | 0 | 0 |
| 5 or more | 11 | 23.4 |  | 3 | 10 |  | 8 | 47.1 |  | 9 | 30 |  | 0 | 0 |  | 2 | 40 |
| Highest education |  |  |  |  |  |  |  |  |  |  |  |  |  |  |  |  |  |
| Less than College | 17 | 34.7 |  | 0 | 0 |  | 17 | 100 |  | 11 | 36.7 |  | 4 | 30.8 |  | 2 | 33.3 |
| College and above | 32 | 65.3 |  | 32 | 100 |  | 0 | 0 |  | 19 | 63.3 |  | 9 | 69.2 |  | 4 | 66.7 |
| From Migori County |  |  |  |  |  |  |  |  |  |  |  |  |  |  |  |  |  |
| No | 20 | 40.8 |  | 18 | 56.2 |  | 2 | 11.8 |  | 13 | 43.3 |  | 5 | 38.5 |  | 2 | 33.3 |
| Yes | 29 | 59.2 |  | 14 | 43.8 |  | 15 | 88.2 |  | 17 | 56.7 |  | 8 | 61.5 |  | 4 | 66.7 |
| Length of stay in Migori County | |  |  |  |  |  |  |  |  |  |  |  |  |  |  |  |  |
| less than 10 years | 14 | 28.6 |  | 14 | 43.8 |  | 0 | 0 |  | 5 | 16.7 |  | 6 | 46.2 |  | 3 | 50 |
| 10 to 20 years | 11 | 22.4 |  | 7 | 21.9 |  | 4 | 23.5 |  | 9 | 30 |  | 1 | 7.7 |  | 1 | 16.7 |
| More than 20 years | 24 | 49 |  | 11 | 34.4 |  | 13 | 76.5 |  | 16 | 53.3 |  | 6 | 46.2 |  | 2 | 33.3 |
| Years as a provider |  |  |  |  |  |  |  |  |  |  |  |  |  |  |  |  |  |
| 0 to 5 years | 18 | 36.7 |  | 12 | 37.5 |  | 6 | 35.3 |  | 12 | 40 |  | 3 | 23.1 |  | 3 | 50 |
| 6 to 10 years | 13 | 26.5 |  | 7 | 21.9 |  | 6 | 35.3 |  | 4 | 13.3 |  | 7 | 53.8 |  | 2 | 33.3 |
| More than 10 years | 18 | 36.7 |  | 13 | 40.6 |  | 5 | 29.4 |  | 14 | 46.7 |  | 3 | 23.1 |  | 1 | 16.7 |
| Years in facility |  |  |  |  |  |  |  |  |  |  |  |  |  |  |  |  |  |
| 0 to 2 years | 18 | 36.7 |  | 15 | 46.9 |  | 3 | 17.6 |  | 11 | 36.7 |  | 4 | 30.8 |  | 3 | 50 |
| 3 to 6 years | 19 | 38.8 |  | 13 | 40.6 |  | 6 | 35.3 |  | 14 | 46.7 |  | 5 | 38.5 |  | 0 | 0 |
| More than 6 years | 12 | 24.5 |  | 4 | 12.5 |  | 8 | 47.1 |  | 5 | 16.7 |  | 4 | 30.8 |  | 3 | 50 |
| Years in current position |  |  |  |  |  |  |  |  |  |  |  |  |  |  |  |  |  |
| less than 2 years | 24 | 49 |  | 20 | 62.5 |  | 4 | 23.5 |  | 18 | 60 |  | 2 | 15.4 |  | 4 | 66.7 |
| 2 to 4 years | 14 | 28.6 |  | 10 | 31.2 |  | 4 | 23.5 |  | 8 | 26.7 |  | 6 | 46.2 |  | 0 | 0 |
| More than 4 years | 11 | 22.4 |  | 2 | 6.2 |  | 9 | 52.9 |  | 4 | 13.3 |  | 5 | 38.5 |  | 2 | 33.3 |
| Number of days worked per week | | |  |  |  |  |  |  |  |  |  |  |  |  |  |  |  |
| 5 or fewer days | 37 | 77.1 |  | 28 | 90.3 |  | 9 | 52.9 |  | 22 | 73.3 |  | 10 | 83.3 |  | 5 | 83.3 |
| More than 5 days | 11 | 22.9 |  | 3 | 9.7 |  | 8 | 47.1 |  | 8 | 26.7 |  | 2 | 16.7 |  | 1 | 16.7 |
| Number of hours working per day | | |  |  |  |  |  |  |  |  |  |  |  |  |  |  |  |
| 8 or fewer hours | 25 | 52.1 |  | 19 | 61.3 |  | 6 | 35.3 |  | 13 | 43.3 |  | 6 | 50 |  | 6 | 100 |
| 9 to 10 hours | 13 | 27.1 |  | 7 | 22.6 |  | 6 | 35.3 |  | 9 | 30 |  | 4 | 33.3 |  | 0 | 0 |
| More than 10 hours | 10 | 20.8 |  | 5 | 16.1 |  | 5 | 29.4 |  | 8 | 26.7 |  | 2 | 16.7 |  | 0 | 0 |
| Ever had training on how to better interact with patients | | | | | | |  |  |  |  |  |  |  |  |  |  |  |
| No | 44 | 89.8 |  | 27 | 84.4 |  | 17 | 100 |  | 28 | 93.3 |  | 11 | 84.6 |  | 5 | 83.3 |
| Yes | 5 | 10.2 |  | 5 | 15.6 |  | 0 | 0 |  | 2 | 6.7 |  | 2 | 15.4 |  | 1 | 16.7 |
|  |  |  |  |  |  |  |  |  |  |  |  |  |  |  |  |  |  |
| Total | 49 | 100 |  | 32 | 100 |  | 17 | 100 |  | 30 | 100 |  | 13 | 100 |  | 6 | 100 |
