# Supplementary material for: Providers’ perceptions of communication and women’s autonomy during childbirth: a mixed methods study in Kenya
Source: Reprod Health. 2020 Jun 3;17:85. doi: 10.1186/s12978-020-0909-0 (PMC7268432; doi:10.1186/s12978-020-0909-0)
Supplement: Supplementary file 3 — Additional file 3. Providers’ perceptions of extent of communication and autonomy by provider type and facility type. [file 12978_2020_909_MOESM3_ESM.docx]

| **Additional file 3: Providers’ perceptions of extent of communication and autonomy by provider type and facility type (N=49)** | | | | | | | | | | | | | | | | | |
| --- | --- | --- | --- | --- | --- | --- | --- | --- | --- | --- | --- | --- | --- | --- | --- | --- | --- |
|  |  |  |  | Provider type | | | | |  | Facilty type | | | | | | | |
|  | Total | |  | Clinical staff | |  | Support staff | |  | Govt. Hospital | |  | Govt. Health Center | |  | Mission Hospital | |
|  | No. | % |  | No. | % |  | No. | % |  | No. | % |  | No. | % |  | No. | % |
|  |  |  |  |  |  |  |  |  |  |  |  |  |  |  |  |  |  |
| Do you think the doctors, nurses, or other health care providers introduce themselves? | | | | | | | | |  |  |  |  |  |  |  |  |  |
| 0 No, none of them | 28 | 57.1 |  | 14 | 43.8 |  | 14 | 82.4 |  | 17 | 56.7 |  | 7 | 53.8 |  | 4 | 66.7 |
| 1 Yes, a few of them | 13 | 26.5 |  | 11 | 34.4 |  | 2 | 11.8 |  | 9 | 30 |  | 3 | 23.1 |  | 1 | 16.7 |
| 2 Yes, most of them | 4 | 8.2 |  | 4 | 12.5 |  | 0 | 0 |  | 3 | 10 |  | 0 | 0 |  | 1 | 16.7 |
| 3 Yes, all of them | 4 | 8.2 |  | 3 | 9.4 |  | 1 | 5.9 |  | 1 | 3.3 |  | 3 | 23.1 |  | 0 | 0 |
| Do you introduce yourself to the women when you first see them? | | | | | |  |  |  |  |  |  |  |  |  |  |  |  |
| 0 No, never | 16 | 33.3 |  | 7 | 22.6 |  | 9 | 52.9 |  | 8 | 26.7 |  | 5 | 38.5 |  | 3 | 60 |
| 1 Yes, a few times | 20 | 41.7 |  | 15 | 48.4 |  | 5 | 29.4 |  | 15 | 50 |  | 4 | 30.8 |  | 1 | 20 |
| 2 Yes, most of the time | 6 | 12.5 |  | 5 | 16.1 |  | 1 | 5.9 |  | 5 | 16.7 |  | 0 | 0 |  | 1 | 20 |
| 3 Yes, all the time | 6 | 12.5 |  | 4 | 12.9 |  | 2 | 11.8 |  | 2 | 6.7 |  | 4 | 30.8 |  | 0 | 0 |
| Do the doctors, nurses, or other health care providers call women by their name? | | | | | | | |  |  |  |  |  |  |  |  |  |  |
| 0 No, never | 6 | 12.2 |  | 3 | 9.4 |  | 3 | 17.6 |  | 3 | 10 |  | 3 | 23.1 |  | 0 | 0 |
| 1 Yes, a few times | 12 | 24.5 |  | 11 | 34.4 |  | 1 | 5.9 |  | 11 | 36.7 |  | 1 | 7.7 |  | 0 | 0 |
| 2 Yes, most of the time | 19 | 38.8 |  | 10 | 31.2 |  | 9 | 52.9 |  | 11 | 36.7 |  | 5 | 38.5 |  | 3 | 50 |
| 3 Yes, all the time | 12 | 24.5 |  | 8 | 25 |  | 4 | 23.5 |  | 5 | 16.7 |  | 4 | 30.8 |  | 3 | 50 |
| Do you refer to women by their names? |  |  |  |  |  |  |  |  |  |  |  |  |  |  |  |  |  |
| 0 No, never | 5 | 10.4 |  | 1 | 3.1 |  | 4 | 25 |  | 3 | 10 |  | 2 | 16.7 |  | 0 | 0 |
| 1 Yes, a few times | 9 | 18.8 |  | 5 | 15.6 |  | 4 | 25 |  | 6 | 20 |  | 2 | 16.7 |  | 1 | 16.7 |
| 2 Yes, most of the time | 17 | 35.4 |  | 14 | 43.8 |  | 3 | 18.8 |  | 11 | 36.7 |  | 4 | 33.3 |  | 2 | 33.3 |
| 3 Yes, all the time | 17 | 35.4 |  | 12 | 37.5 |  | 5 | 31.2 |  | 10 | 33.3 |  | 4 | 33.3 |  | 3 | 50 |
| (If a clinician): Do you explain to women why you are doing examinations or procedures on them? | | | | | | | | | |  |  |  |  |  |  |  |  |
| 0 No, never |  |  |  |  |  |  |  |  |  |  |  |  |  |  |  |  |  |
| 1 Yes, a few times |  |  |  |  |  |  |  |  |  |  |  |  |  |  |  |  |  |
| 2 Yes, most of the time | 10 | 33.3 |  | 10 | 33.3 |  |  |  |  | 6 | 31.6 |  | 2 | 28.6 |  | 2 | 50 |
| 3 Yes, all the time | 20 | 66.7 |  | 20 | 66.7 |  |  |  |  | 13 | 68.4 |  | 5 | 71.4 |  | 2 | 50 |
| (If a clinician): Do you explain to women why you are giving them medicines? | | | | | | |  |  |  |  |  |  |  |  |  |  |  |
| 0 No, never | 1 | 3.3 |  | 1 | 3.3 |  |  |  |  | 0 | 0 |  | 0 | 0 |  | 1 | 25 |
| 1 Yes, a few times | 2 | 6.7 |  | 2 | 6.7 |  |  |  |  | 2 | 11.1 |  | 0 | 0 |  | 0 | 0 |
| 2 Yes, most of the time | 6 | 20 |  | 6 | 20 |  |  |  |  | 3 | 16.7 |  | 2 | 25 |  | 1 | 25 |
| 3 Yes, all the time | 21 | 70 |  | 21 | 70 |  |  |  |  | 13 | 72.2 |  | 6 | 75 |  | 2 | 50 |
| Do the doctors, nurses or other staff at the facility ask women’s permission/consent before examinations and procedures? | | | | | | | | | | | | | |  |  |  |  |
| 0 No, never | 4 | 8.2 |  | 2 | 6.2 |  | 2 | 11.8 |  | 2 | 6.7 |  | 1 | 7.7 |  | 1 | 16.7 |
| 1 Yes, a few times | 4 | 8.2 |  | 2 | 6.2 |  | 2 | 11.8 |  | 2 | 6.7 |  | 2 | 15.4 |  | 0 | 0 |
| 2 Yes, most of the time | 26 | 53.1 |  | 18 | 56.2 |  | 8 | 47.1 |  | 16 | 53.3 |  | 5 | 38.5 |  | 5 | 83.3 |
| 3 Yes, all the time | 13 | 26.5 |  | 10 | 31.2 |  | 3 | 17.6 |  | 9 | 30 |  | 4 | 30.8 |  | 0 | 0 |
| 8 Don’t know | 2 | 4.1 |  | 0 | 0 |  | 2 | 11.8 |  | 1 | 3.3 |  | 1 | 7.7 |  | 0 | 0 |
| In your experience, are women or families given information about their care? | | | | | | |  |  |  |  |  |  |  |  |  |  |  |
| 0 No, never |  |  |  |  |  |  |  |  |  |  |  |  |  |  |  |  |  |
| 1 Yes, a few times | 7 | 14.3 |  | 5 | 15.6 |  | 2 | 11.8 |  | 5 | 16.7 |  | 2 | 15.4 |  | 0 | 0 |
| 2 Yes, most of the time | 27 | 55.1 |  | 16 | 50 |  | 11 | 64.7 |  | 18 | 60 |  | 5 | 38.5 |  | 4 | 66.7 |
| 3 Yes, all the time | 15 | 30.6 |  | 11 | 34.4 |  | 4 | 23.5 |  | 7 | 23.3 |  | 6 | 46.2 |  | 2 | 33.3 |
| Do you feel women can ask the doctors, nurses or other staff at the facility any questions they have? | | | | | | | | | | |  |  |  |  |  |  |  |
| 0 No, never |  |  |  |  |  |  |  |  |  |  |  |  |  |  |  |  |  |
| 1 Yes, a few times | 8 | 16.3 |  | 5 | 15.6 |  | 3 | 17.6 |  | 4 | 13.3 |  | 3 | 23.1 |  | 1 | 16.7 |
| 2 Yes, most of the time | 20 | 40.8 |  | 11 | 34.4 |  | 9 | 52.9 |  | 13 | 43.3 |  | 3 | 23.1 |  | 4 | 66.7 |
| 3 Yes, all the time | 20 | 40.8 |  | 16 | 50 |  | 4 | 23.5 |  | 12 | 40 |  | 7 | 53.8 |  | 1 | 16.7 |
| 8 Don’t know | 1 | 2 |  | 0 | 0 |  | 1 | 5.9 |  | 1 | 3.3 |  | 0 | 0 |  | 0 | 0 |
| Do you feel women can ask you any questions they have? | | | |  |  |  |  |  |  |  |  |  |  |  |  |  |  |
| 0 No, never |  |  |  |  |  |  |  |  |  |  |  |  |  |  |  |  |  |
| 1 Yes, a few times | 4 | 8.2 |  | 2 | 6.2 |  | 2 | 11.8 |  | 3 | 10 |  | 1 | 7.7 |  | 0 | 0 |
| 2 Yes, most of the time | 24 | 49 |  | 15 | 46.9 |  | 9 | 52.9 |  | 16 | 53.3 |  | 4 | 30.8 |  | 4 | 66.7 |
| 3 Yes, all the time | 21 | 42.9 |  | 15 | 46.9 |  | 6 | 35.3 |  | 11 | 36.7 |  | 8 | 61.5 |  | 2 | 33.3 |
| Do the doctors, nurses or other staff at the facility answer questions family have? | | | | | | |  |  |  |  |  |  |  |  |  |  |  |
| 0 No, never |  |  |  |  |  |  |  |  |  |  |  |  |  |  |  |  |  |
| 1 Yes, a few times | 3 | 6.1 |  | 3 | 9.4 |  | 0 | 0 |  | 2 | 6.7 |  | 0 | 0 |  | 1 | 16.7 |
| 2 Yes, most of the time | 32 | 65.3 |  | 21 | 65.6 |  | 11 | 64.7 |  | 23 | 76.7 |  | 7 | 53.8 |  | 2 | 33.3 |
| 3 Yes, all the time | 14 | 28.6 |  | 8 | 25 |  | 6 | 35.3 |  | 5 | 16.7 |  | 6 | 46.2 |  | 3 | 50 |
| Do the doctors, nurses or other staff at the facility speak to women in a language they understand? | | | | | | | | | | |  |  |  |  |  |  |  |
| 0 No, never |  |  |  |  |  |  |  |  |  |  |  |  |  |  |  |  |  |
| 1 Yes, a few times | 4 | 8.2 |  | 3 | 9.4 |  | 1 | 5.9 |  | 2 | 6.7 |  | 2 | 15.4 |  | 0 | 0 |
| 2 Yes, most of the time | 19 | 38.8 |  | 13 | 40.6 |  | 6 | 35.3 |  | 12 | 40 |  | 5 | 38.5 |  | 2 | 33.3 |
| 3 Yes, all the time | 26 | 53.1 |  | 16 | 50 |  | 10 | 58.8 |  | 16 | 53.3 |  | 6 | 46.2 |  | 4 | 66.7 |
| Do you feel like the doctors, nurses or other staff at the facility involve women in decisions about their care? | | | | | | |  |  |  |  |  |  |  |  |  |  |  |
| 0 No, never | 1 | 2 |  | 0 | 0 |  | 1 | 5.9 |  | 0 | 0 |  | 1 | 7.7 |  | 0 | 0 |
| 1 Yes, a few times | 4 | 8.2 |  | 4 | 12.5 |  | 0 | 0 |  | 4 | 13.3 |  | 0 | 0 |  | 0 | 0 |
| 2 Yes, most of the time | 31 | 63.3 |  | 19 | 59.4 |  | 12 | 70.6 |  | 19 | 63.3 |  | 8 | 61.5 |  | 4 | 66.7 |
| 3 Yes, all the time | 13 | 26.5 |  | 9 | 28.1 |  | 4 | 23.5 |  | 7 | 23.3 |  | 4 | 30.8 |  | 2 | 33.3 |
| During the delivery, do you feel like women are able to be in the position of their choice? | | | | | | |  |  |  |  |  |  |  |  |  |  |  |
| 0 No, never | 18 | 37.5 |  | 10 | 32.3 |  | 8 | 47.1 |  | 13 | 43.3 |  | 2 | 16.7 |  | 3 | 50 |
| 1 Yes, a few times | 13 | 27.1 |  | 8 | 25.8 |  | 5 | 29.4 |  | 10 | 33.3 |  | 2 | 16.7 |  | 1 | 16.7 |
| 2 Yes, most of the time | 12 | 25 |  | 9 | 29 |  | 3 | 17.6 |  | 5 | 16.7 |  | 6 | 50 |  | 1 | 16.7 |
| 3 Yes, all the time | 4 | 8.3 |  | 4 | 12.9 |  | 0 | 0 |  | 1 | 3.3 |  | 2 | 16.7 |  | 1 | 16.7 |
| 8 Don’t know | 1 | 2.1 |  | 0 | 0 |  | 1 | 5.9 |  | 1 | 3.3 |  | 0 | 0 |  | 0 | 0 |
| Do you think it is important to introduce yourself to patients? | | | |  |  |  |  |  |  |  |  |  |  |  |  |  |  |
| 0 No, not important | 2 | 4.1 |  | 0 | 0 |  | 2 | 11.8 |  | 0 | 0 |  | 2 | 15.4 |  | 0 | 0 |
| 1 Yes, somewhat important | 5 | 10.2 |  | 2 | 6.2 |  | 3 | 17.6 |  | 2 | 6.7 |  | 1 | 7.7 |  | 2 | 33.3 |
| 2 Yes, very important | 42 | 85.7 |  | 30 | 93.8 |  | 12 | 70.6 |  | 28 | 93.3 |  | 10 | 76.9 |  | 4 | 66.7 |
| Do you think it is important to call women by their names? | | | |  |  |  |  |  |  |  |  |  |  |  |  |  |  |
| 1 Yes, somewhat important | 10 | 20.8 |  | 2 | 6.2 |  | 8 | 50 |  | 6 | 20.7 |  | 3 | 23.1 |  | 1 | 16.7 |
| 2 Yes, very important | 38 | 79.2 |  | 30 | 93.8 |  | 8 | 50 |  | 23 | 79.3 |  | 10 | 76.9 |  | 5 | 83.3 |
| Do you feel it important for doctors, nurses or other staff at the facility to ask permission or consent before procedures? | | | | | | | | | | | | | |  |  |  |  |
| 0 No, not important | 1 | 2 |  | 0 | 0 |  | 1 | 5.9 |  | 0 | 0 |  | 1 | 7.7 |  | 0 | 0 |
| 1 Yes, somewhat important | 7 | 14.3 |  | 2 | 6.2 |  | 5 | 29.4 |  | 4 | 13.3 |  | 3 | 23.1 |  | 0 | 0 |
| 2 Yes, very important | 41 | 83.7 |  | 30 | 93.8 |  | 11 | 64.7 |  | 26 | 86.7 |  | 9 | 69.2 |  | 6 | 100 |
| Do you think is important to involve women or their families in decisions about their care? | | | | | | | | |  |  |  |  |  |  |  |  |  |
| 1 Yes, somewhat important | 7 | 14.6 |  | 3 | 9.4 |  | 4 | 25 |  | 4 | 13.8 |  | 3 | 23.1 |  | 0 | 0 |
| 2 Yes, very important | 41 | 85.4 |  | 29 | 90.6 |  | 12 | 75 |  | 25 | 86.2 |  | 10 | 76.9 |  | 6 | 100 |
|  |  |  |  |  |  |  |  |  |  |  |  |  |  |  |  |  |  |
| Total | 49 | 100 |  | 32 | 100 |  | 17 | 100 |  | 30 | 100 |  | 13 | 100 |  | 6 | 100 |
